# Preliminary associations between pet ownership and mental health in youth with diabetes

**DOI:** 10.3389/fcdhc.2026.1760110

**Published:** 2026-03-16

**Authors:** Noa R. Mills, Erin K. King, Megan K. Mueller

**Affiliations:** 1 Department of Occupational Therapy at Tufts University, Medford, MA, United States; 2 Center for Animals and Public Policy, Cummings School of Veterinary Medicine at Tufts University, North Grafton, MA, United States

**Keywords:** companion animals, diabetes, pets, stress, youth

## Abstract

**Introduction:**

Youth with diabetes are at a higher risk for mental health challenges. Despite this awareness, there is much to learn how factors of a child’s environment, such as pet ownership, may promote better diabetes-related health outcomes. This study assessed if pet ownership in diabetic youth was associated with anxiety/depression, parental stress, and A1C, as well as if geographic prevalence of diabetes differed by pet ownership status.

**Methods:**

Data were obtained from the Adolescent Brain Cognitive Development (ABCD) Study, including participants with pet ownership data (*n* = 9,802). Primary analyses were conducted with a subsample of youth with diabetes (*n* = 80).

**Results:**

There were no statistically significant relationships between pet ownership and anxiety/depression or parental stress. A1C levels among pet owners and non pet owners were explored (*n* = 10). In the full sample (*n* = 9,802), there was a significant difference between pet owners and non-pet owners on geographical diabetes prevalence where pet owners were more likely to live in communities with lower rates of diabetes.

**Conclusions:**

Pet ownership was not associated with mental health in families with diabetes. Preliminary analyses indicated the potential for an association between A1C and pet ownership as well as geographic prevalence of diabetes and living with a pet.

## 1 Introduction

Diabetes is a serious health concern that has increased exponentially among all ages, sexes, races, and socio-economic groups throughout the world in recent years (1). The complex and demanding treatment regimen children with diabetes undergo puts them at a higher risk for psychological disorders (2). Furthermore, parents of children with diabetes also face a greater risk of depression due to increased fear and management of medical challenges (3). Despite awareness of the stress that childhood diabetes can have on both the child and the parents, there is still much more research needed to learn how factors of a child’s environment may promote better diabetes-related health outcomes. One of these environmental factors may be pet ownership. Pet ownership is very common in the United States, with over 65% of families having at least one pet (4); therefore, having a pet is a potentially common source of support for children with diabetes.

Numerous studies demonstrate a relevant relationship between diabetes and mental health disorders. Specifically, youth with Type 1 Diabetes (T1DM) have a 2–3-fold increased risk for psychiatric disorders (5). Overall, youth with T1DM are at higher risk for depression, anxiety, and other mental health challenges (6–8). This high prevalence of psychological challenges in children with diabetes suggests a need for supporting strategies within this population to assist youth in managing their physical and mental health.

Mental health challenges affect not only diabetic children, but also their families. Managing a child’s diabetes requires both the parent and the child to adhere to a certain protocol and routine to ensure the child’s health and safety. One of the strongest risk factors for depressive disorders in adolescents with T1DM is maternal depression, which occurs in about one-third of mothers of youth with T1DM (2). Previous research has shown a correlation between general stress of the parent and high levels of child depressive symptoms, poorer self-care behavior, and worse glycemic control (9). More research needs to be conducted to show what factors in a diabetic child’s life can have a positive impact on the child and their family’s life to mitigate the effects of stress.

### 1.1 Pet ownership, mental health, and diabetes

There is growing evidence that family pets in particular encourage positive interactions that favor closeness, sharing, and empathy resulting in more prosocial relational models (10–12). Moreover, pets (specifically dogs) have been found to be supportive for children during stressful situations due to lowering their cortisol levels, a stress hormone which impacts both mental health and A1C in the bloodstream (13). If pet ownership can positively impact social-emotional development and cortisol levels in children more generally, one may draw the parallel that it may also help alleviate some of the risk of anxiety and depression in children with diabetes.

Similarly, taking care of a pet can positively impact children with conditions such as anxiety, depression, and socioemotional challenges (14). Research has also shown that having a dog led to a decreased likelihood of general anxiety and children with pets tend to present better emotional well-being and reduced feelings of loneliness and depressive symptoms (15).

Two studies have explored the correlation between pet ownership and the physical effects of diabetes in children via A1C levels (16, 17). One of the studies found that children with A1C values below the American Diabetes Association target were more likely to have taken responsibility for a household pet than those with A1C values above the target (17). Another study used an experimental design and found the participants given the responsibility of a goldfish exhibited a statistically significant decrease in A1C level compared with their peers in the control group, who had an increase in A1C level (16). These findings point to a potential role for pets in supporting youth with diabetes and their families.

### 1.2 Current study

This study aims to explore associations between living with pets and indicators of mental health of diabetic children and their parents using a geographically diverse sample of families living in the United States. The goal of this study was to assess if pet owners and non-pet owners differed in mental health outcomes with regard to youth anxiety/depression and parental stress levels. We hypothesized that diabetic children who own pets would report lower levels of anxiety and depression and lower parental stress levels compared their non-pet-owning peers. This study also had two exploratory, descriptive analyses, one with a smaller subsample, assessing A1C in relation to pet ownership, and a second assessing if pet ownership was associated with community diabetes prevalence.

## 2 Methods

Data used in this study were obtained from The Adolescent Brain Cognitive Development (ABCD) Study. The ABCD Study is a large, long-term study of brain development and child health in the United States funded by the National Institutes of Health (NIH). Researchers are tracking biological and behavioral development through adolescence into young adulthood. For more information about the ABCD Study, please visit the ABCD Study website. The analyses presented in this paper were reviewed by the Social, Behavioral, and Educational Research (SBER) IRB at Tufts University and were determined not human research due to the use of secondary de-identified data (reference number: STUDY00003507). The analyses in this paper represent a cross-sectional cohort analysis.

### 2.1 Participants and procedures

The ABCD Research Consortium consists of 21 research sites across the country, which invited 11,880 children between the ages of 9 and 10 to participate. Youth and their parents are included in the study for 10 years, until they are 19 or 20 years old. Recruitment areas were determined by locations of 21 study sites (catchment areas), which closely matched the sociodemographic composition of the US population. All adult participants (parents/guardians) provided written informed consent, and all youth provided written assent along with permission from a parent/guardian.

This analysis included inclusion criteria beyond the general criteria for the ABCD Study to focus on children living with diabetes. Youth were included in this analysis if their parent reported their child ever being seen by the doctor for diabetes at the baseline, 2-year, or 3-year follow up data collection points (collapsed across years during the time period of 2016-2023, depending on time of enrollment of each youth/family) (*n =* 80). Outcome measures were derived from the 3-year follow up data from the ABCD Study Data release 5.1 (data released in 2023). The dependent variables were measured using self-reported survey data that was taken by the parents (*n =* 80) as well as a blood sample to measure A1C (*n* = 10). Baseline data were used for demographic information. In addition, we conducted an exploratory analysis of residential diabetes history which utilized any participant in the ABCD Study sample with data on pet ownership status (*n* = 9,802).

### 2.2 Measures

#### 2.2.1 Demographics and pet ownership

Youth participants reported their age (recorded in months, re-coded to years) and gender (male, female, trans male, trans female, gender queer). Participants’ parents/guardians were also asked to identify their child’s racial and ethnic identity. Parents/guardians reported their highest level of education obtained in 20 categories, ranging from never attended/kindergarten only through professional/doctoral degree, which was recoded into three categories for analysis: high school education or less, some college (through bachelor’s degree), graduate degree and higher. Combined family income had eight different options, beginning from ≤$5,000 to ≥$200,000. This demographic variable was categorized into 3 different groups: <$49,999, $50,000-$99,999, and >$100,000. Lastly, parent’s marital status included married, divorced, separated, never married, or living with partner. See **Table 1** for demographic data stratified by pet ownership status.

**Table 1:** Diabetic sample demographics stratified by pet ownership status.

Demographic variables	Total sample *n* = 80	No pet *n* = 23	Pet owner *n* = 57	Missing
*Age (years)*	*M* (SD) 12.88 (0.65)	*M* (SD) 13.03 (0.59)	*M* (SD) 12.82 (0.66)	
*Gender*	*n* (%)	*n* (%)	*n* (%)	0 (0%)
Female	43 (53.8%)	7 (30.4%)	36 (63.2%)	
Male	37 (46.3%)	16 (69.6%)	21 (36.8%)	
Race/Ethnicity*				1 (1.3%)
White	46 (57.5%)	6 (26.1%)	40 (70.2%)	
Black/African American	32 (40%)	16 (69.6%)	16 (28.1%)	
Chinese	1 (1.3%)	0 (0.0%)	1 (1.8%)	
Filipino	1 (1.3%)	1 (4.3%)	0 (0.0%)	
Indigenous	6 (7.5%)	1 (4.3%)	5 (8.8%)	
Other Race	7 (8.8%)	3 (13.0%)	4 (7.0%)	
Hispanic	16 (20%)	4 (17.4%)	12 (21.1%)	1 (1.3%)
*Parent Education*				1 (1.3%)
High School or less	25 (31.3%)	9 (39.1%)	16 (28.0%)	
Any undergraduate	40 (50.0%)	10 (43.5%)	30 (52.6%)	
Graduate degree	14 (17.5%)	3 (13.0%)	11 (19.3%)	
*Combined Family Income*				6 (7.5%)
≤ $49,999	32 (40.0%)	11 (47.8%)	21 (36.8%)	
$50,000-99,999	24 (30.0%)	7 (30.4%)	17 (29.8%)	
≥ $100,000	18 (22.5%)	4 (17.4%)	14 (24.5%)	
*Parent Marital Status*				
Married	42 (52.5%)	9 (39.1%)	33 (57.9%)	2 (2.5%)
Divorced	10 (12.5%)	2 (8.7%)	8 (14.0%)	
Separated	5 (6.3%)	2 (8.7%)	3 (5.3%)	
Never Married	18 (22.5%)	7 (30.4%)	11 (19.3%)	
Living with Partner	3 (3.8%)	1 (4.3%)	2 (3.5%)	

*Participants were able to select more than one racial/ethnic identity; percentages may add up to more than 100%. No participants in this subsample identified as Alaska Native, Native Hawaiian, Guamanian, Samoan, Other Pacific Islander, Asian Indian, Japanese, Korean, Vietnamese, or Other Asian.

Pet ownership was measured by asking youth participants if they lived with a pet in the family, and if so, what kind of pet(s) did they have (dog, cat, horse, fish, small animal [e.g., rabbit, hamster, bird], or other). Out of the total 80 participants in the study, 57 participants (70.37% of the sample) reported having a pet, which is similar to pet ownership rates for the whole ABCD Study sample (approximately 76% pet owners; 18). Due to the small sample size, pet ownership was recorded as dichotomous - either pet owner or non-pet owners - and not analyzed by species.

#### 2.2.2 Parent-reported youth anxiety/depression

Anxiety and depression were measured using The Child Behavioral Checklist (CBCL), which is a parent rated scale. The CBCL is used to detect emotional and behavioral problems in children and adolescents across the past 6 months. The parents answered questions that are rated on a 3-point rating scale (not true, somewhat/sometimes true, and very true/often true). For this study, we used the Anxious/Depressed (CBCL-A/D) subscale, which includes 13 of the 113 total items. Higher scores on the CBCL-A/D indicate greater challenges with anxiety and depression. The CBCL t-score can be categorized into non-clinical symptoms (≤59), at risk for problem behavior (60-64), and clinical symptoms (>65) (19). For this analysis, due to the small sample size, we re-coded the variable into two categories, non-clinical symptoms (≤59), and at risk or clinical symptoms (≥60).

#### 2.2.3 Parental stress

Parental stress was measured using the Perceived Stress Scale (PSS-10). The PSS-10 is a 10-item questionnaire originally developed by Cohen et al. (20) which is widely used to assess stress levels. It evaluates the degree to which an individual has perceived life as unpredictable, uncontrollable, and overloading over the previous month. The questions in this scale ask about the participant’s feelings and thoughts during the last month. In each question, it is asked to indicate how often they personally felt or thought a certain way. The scale is from 0 to 4 (0=never, 1=almost never, 2=sometimes, 3=fairly often, 4=very often). PSS scores are obtained by reversing responses to the four positively stated items and then summing across all scale items to create an overall stress index with higher scores indicating higher stress.

#### 2.2.4 A1C

A1C measurements are a biospecimen that was gathered by blood samples taken by the researchers at one of the 21 research sites across the ABCD Study. These blood samples were analyzed using whole blood analysis. Data on A1C levels were only available for 10 participants at the time of analysis, thus these analyses are exploratory.

#### 2.2.5 Residential diabetes prevalence

Residential prevalence of diagnosed diabetes among adults aged >=18 years within the community is a linked variable provided ABCD Study dataset. This variable is derived from the Centers for Disease Control 2020 PLACES data release, and includes residential data at the Census tract level (21). The residential diabetes prevalence variable represents the prevalence of diabetes in the community that the ABCD Study participant lives in.

### 2.3 Data analysis

All analyses were conducted using SPSS software version 29 (22). Within the diabetic sample, descriptive statistics and frequencies are reported for pet ownership and demographic variables. Given the small amount of missing data in this dataset, missing data were treated using listwise deletion. Data were assessed for normality prior to statistical analysis. For the parental stress, an independent samples t-test was used to assess differences between pet owners and non-pet owners, with a *p* value of <.05 indicating significance. The anxiety/depression variable (CBCL-A/D) was not normally distributed and demonstrated a bimodal distribution. Therefore, we used categorization guidance to re-code the CBCL-A/D into two categories representing low risk and higher risk for anxiety/depression (see Measures section for additional details). Chi-square analyses were used to assess differences in the CBCL-A/D between families with and without pets.

As an exploratory analysis, A1C levels were also analyzed to identify if pet ownership was associated with differences in glycemic control. However, within the diabetic population in the ABCD study (*n* = 80), only 10 participants had blood marker data (pet ownership: *n* = 6, non-pet owner: *n* = 4), thus descriptive statistics are presented. We used an independent samples t-test to assess differences in diabetes prevalence between the pet owner group (*n* = 7,504) and the non-pet owner group (*n* = 2,298).

## 3 Results

**Table 1** reports demographic variables, stratified by pet ownership status, indicating that there are variations in sociodemographic characteristics of families who have pets and those who do not, within the subsample of youth with diabetes. Independent samples t-test results indicated that pet ownership was not significantly associated with parental stress (t[75]=-.0.63, *p* = .53), and the effect size was small (Cohen’s *d* = .16), suggesting owning a pet does not significantly predict parental stress for families with diabetic children. Pet owners had average stress scores of 14.54 (*SD* = 6.71) compared to 15.59 in non-pet owners (*SD* = 6.38).

Chi-square analyses indicated there was no significant difference in the proportion of low and high anxiety CBCL-A/D scores between pet owners and non-pet owners (χ^2^[1]=0.62, *p>.*05). Within participants who owned pets, 76.4% (*n* = 33) reported low anxiety, compared to 70.59% (*n=*12) among participants without pets., but no conclusions can be made from these results due to the lack of statistical significance and small sample size.

There was a very small sample size for participants who had A1C level data (*n =* 10). The average A1C level for pet owners was 5.27 (*n* = 6; *SD* = 0.43), as compared to 5.68 for non-pet owners (*n* = 4; *M* = 5.68; *SD* = 0.73). These results indicate that the relationship between pet ownership and A1C levels should be explored in a larger sample.

There was a significant difference between pet owners and non-pet owners on geographical population-based diabetes prevalence, with a small effect size (*t*[3190.22]=-12.51, *p* < 0.001, Cohen’s *d* = 0.34). The average prevalence of diabetes for the pet ownership group was lower (*M=*9.46; *SD=*3.30) compared to non-pet owners (*M=*10.67; *SD=*4.25). The t-test results indicate that pet owners live in communities with lower rates of diabetes than non-pet owners.

## 4 Discussion

Overall, the results of this study did not support the hypothesis that pet ownership in families with diabetic children is significantly associated with their levels of anxiety/depression or parental stress. There were no significant differences for any of the variables, however, the association between pet ownership and A1C demonstrated differences with a medium effect size, which indicates these relationships may be worth exploring further with a larger sample size.

### 4.1 Anxiety/depression and parental stress

Results indicated no significant differences between pet owners and non-pet owners on anxiety/depression and parental stress. These findings differ from some previous research indicating that pet ownership can have a positive relationship in children’s mental health and parental stress, though is representative of some of the broader work in this area showing mixed findings (10–12, 14). One explanation for this finding is that living with a pet may not buffer stress and anxiety for a family with a child with diabetes, or it may also be a function of the relatively small sample size for this study. Research with larger sample sizes should explore these associations in more detail.

### 4.2 A1C exploratory analyses

In our exploratory analysis of A1C levels, we found that the A1C levels in our study of both pet and non-pet owners were within the normal range of 4.5% to 5.7%. However, the pet ownership group descriptively had a lower average A1C level compared to the non-pet owners. While interpreting these results is limited by the small sample size, they indicate potential alignment with prior research finding that diabetic children with pets had better control and lower A1C measurement than their non-pet ownership counterparts in both retrospective design and experimental design (16, 17). In both studies, responsibility and self-efficacy are seen as beneficial aspect of having a pet that support the child’s own management in their glycemic control. However, the exploratory analyses in this study were conducted with a very small sample (*n =* 10) and should not be over-interpreted, but the results suggest that further research with larger sample sizes is warranted.

### 4.3 Diabetic population density and pet ownership

According to our findings, pet owners in the overall ABCD Study sample live in areas with lower percentages of diabetes prevalence compared to non-pet owners. There may be numerous reasons for this correlation that are not causal in nature. Forxexample, diabetes disproportionally affects individuals identifying as Black or Hispanic (23). These patterns correlate with similar patterns in pet ownership; prior research has shown that pet ownership is highest among White households and Black/African American households make up the lowest percentage of households owning pets (24). Thus, Black households are both disproportionately diabetic and the least likely to own a pet; these two occurrences may not be related but are correlational. It may be that there are systemic inequities that impact families’ access to both pets and healthcare services.

Although pet ownership may be positive for some families, pets are also not cost-neutral. As diabetes is more prevalent in lower income communities (23), pet ownership may be related to income as well. Diabetes requires additional medical care and financial pressures (4) which may mean individuals and families are not able to afford a pet and the associated costs. Since health care costs are greater, the cost of a pet may not be feasible or may increase financial distress. This high cost for pets may be burdensome to families who are already managing high healthcare costs. Overall, exploratory findings in our study indicate that systematic inequities in access to healthcare and pets should be explored in more detail.

### 4.4 Limitations

As the dataset analyzed in this study was only a small sample from a larger dataset, the generalizability is limited and future research with larger sample sizes may more fully elucidate the relationship between pet ownership and health outcomes for youth with diabetes. Furthermore, the sample size was too small to perform more nuanced modeling techniques that controlled for demographic covariates that could potentially be cofounding variables (including urbanicity of housing location), which is a substantial limitation based on prior research documenting the role of covariates in pet-related health outcomes. The primary limitations in the study were the small sample size within the ABCD dataset with diagnosed diabetes, the unknown information of if the children were type 1 or type 2 diabetics, the disproportionate size of the two groups, and the very limited number of participants who had their A1C measurements taken. Furthermore, there were measurement limitations, with limited information about the construct validity of the CBCL and the PSS in measuring mental health outcomes that are relevant and associated with pet ownership. The prevalence of diabetes was measured as an ecological variable, so the results cannot be extrapolated to the individual level.

Future studies should focus on how different life contexts including pet ownership could benefit the child and the parent both mentally and physically. Ultimately, this study contributes to the current literature by suggesting that pet ownership may be worth exploring further as a factor supporting diabetic children in their A1C and future research is required for fully understanding the role of pets in supporting anxiety/depression, emotional regulation, and parental stress. Additionally, our findings emphasize the importance demographics have on the diabetic population for either pet ownership or another intervention but will require a larger data pool.

### 4.5 Conclusion

This study is the first to use a nationally representative dataset assessing the relationship between pet ownership and diabetic children’s mental health and parental stress. Although the results suggest that pet ownership is not significantly associated with measures of well-being in diabetic children used in this study, some of the findings suggest future directions for research in this area.

## Data Availability

Publicly available datasets were analyzed in this study. This data can be found here: DOI: 10.15154/z563-zd24.
